# A Multilevel Probabilistic Cerenkov Luminescence Tomography Reconstruction Framework Based on Energy Distribution Density Region Scaling

**DOI:** 10.3389/fonc.2021.751055

**Published:** 2021-10-22

**Authors:** Xiao Wei, Hongbo Guo, Jingjing Yu, Xuelei He, Huangjian Yi, Yuqing Hou, Xiaowei He

**Affiliations:** ^1^ School of Information and Technology, Northwest University, Xi’an, China; ^2^ Xi’an Key Laboratory of Radiomics and Intelligent Perception, Northwest University, Xi’an, China; ^3^ School of Physics and Information Technology, Shaanxi Normal University, Xi’an, China

**Keywords:** Cerenkov luminescence tomography, probabilistic, covariance, inverse problem, region of interest

## Abstract

Cerenkov luminescence tomography (CLT) is a promising non-invasive optical imaging method with three-dimensional semiquantitative *in vivo* imaging capability. However, CLT itself relies on Cerenkov radiation, a low-intensity radiation, making CLT reconstruction more challenging than other imaging modalities. In order to solve the ill-posed inverse problem of CLT imaging, some numerical optimization or regularization methods need to be applied. However, in commonly used methods for solving inverse problems, parameter selection significantly influences the results. Therefore, this paper proposed a probabilistic energy distribution density region scaling (P-EDDRS) framework. In this framework, multiple reconstruction iterations are performed, and the Cerenkov source distribution of each reconstruction is treated as random variables. According to the spatial energy distribution density, the new region of interest (ROI) is solved. The size of the region required for the next operation was determined dynamically by combining the intensity characteristics. In addition, each reconstruction source distribution is given a probability weight value, and the prior probability in the subsequent reconstruction is refreshed. Last, all the reconstruction source distributions are weighted with the corresponding probability weights to get the final Cerenkov source distribution. To evaluate the performance of the P-EDDRS framework in CLT, this article performed numerical simulation, *in vivo* pseudotumor model mouse experiment, and breast cancer mouse experiment. Experimental results show that this reconstruction framework has better positioning accuracy and shape recovery ability and can optimize the reconstruction effect of multiple algorithms on CLT.

## 1 Introduction

*Cerenkov radiation* (CR) is a physical phenomenon that occurs when a particle traveling through an object travels faster than the speed of light in the medium (1). The first radiographic imaging using CR was made in 2009 and is known as Cerenkov luminescence imaging (CLI) (2). Since its inception, CLI has been widely used in surgical guidance, drug development, endoscopic imaging, tumor detection, and other fields (3–7). The most significant advantage of CLI over other optical imaging methods is that it can use many approved radioactive sources for clinical imaging (8–10). However, CLI is a planar imaging method and cannot obtain the depth information and three-dimensional (3D) distribution of radioactive sources. Therefore, a new optical imaging method, Cerenkov luminescence tomography (CLT), combined with CLI and 3D anatomical imaging modality, has been developed. Compared with CLI, CLT can obtain the internal and external contour or boundary of biological tissues with the 3D anatomical imaging modality and determine the 3D spatial distribution of radioactive source in biological tissues (11–15).

However, the Cerenkov photon energy mainly concentrated in the short-wavelength band with a high scattering in biological tissues, which leads to the difficulty for CLT reconstruction (16). Therefore, compared with bioluminescence tomography (BLT) and fluorescence molecular tomography (FMT), CLT requires more prior information constraints and more robust algorithms to optimize its solution (17, 18). Therefore, researchers have carried out a series of work in algorithms and feasible region constraints for CLT reconstruction.

From the algorithm’s point of view, improving the current reconstruction algorithm is a research focus, such as L_1_ norm regularization (19), L_2_ norm regularization (11), LP_(0<p<1)_ norm regularization, and other regularization methods (12), that has been used in the CLT field. Although these regularization methods can be applied to CLT, they depend on the selection of regularization parameters. In addition to regularization algorithms, there are other areas of inverse problem algorithms; non-regularization methods such as orthogonal matching pursuit (OMP) (20) do not require regularization parameter selection, but they have poor performance in CLT reconstruction.

From the perspective of the feasible region, some feasible region iterative methods, such as iterative shrinking permissible region (ISPR) (21), three-way decision (TWD) (22), and feature extraction from the autoencoder (3), have already been applied to optical molecular imaging. These methods constrain the solution space by regions of interest (ROIs) to obtain better reconstruction results. However, the current feasible region method has the following shortcomings: first, the feasible region only shrinks and may lose some nodes; second, the final reconstruction results are only affected by the previous one, ignoring the intermediate process; and third, errors in the iterative process will be passed to the subsequent iterative methods, leading to polluted results. In general, most of the reconstruction algorithms or strategies are limited by the weak signal intensity of CR, which leads to the difficulty of solving the inverse problem, or the parameter tuning of the reconstruction algorithm itself needs to be carried out at a high cost. From what has been discussed above, many inverse problem algorithms or feasible region strategies are challenging to apply to the CLT field.

To optimize the feasible region method in CLT and improve the performance of traditional algorithms in CLT reconstruction, this paper proposed a multilevel probabilistic energy distribution density region scaling (P-EDDRS) framework for CLT. In this framework, L_2_ norm error rate and cosine similarity were used to evaluate the quality of each iteration reconstruction result, and normalized weight was assigned to each reconstruction result according to the evaluation. This normalized weight represents the probability that the corresponding iteration result is the final result. By this weight, the initial iteration reconstruction result becomes a probabilistic result. Then, the probabilistic result is regarded as random variables distributed in 3D space, and ROI for the next iteration is divided according to the distribution density of these random variables. Besides, to stabilize the rate of ROI change, the formula proposed by Naser et al. was introduced (23). After several iterations, all the initial reconstruction source distribution and the corresponding normalized weight are weighted to get the final Cerenkov source distribution.

To evaluate the performance of the P-EDDRS frame in CLT, several groups of numerical simulations and *in vivo* experiments were implemented. The optimized Lasso and Least Square QR-factorization (LassoLSQR) algorithm based on L_1_ norm regularization (24), Tikhonov regularization algorithm based on L_2_ norm regularization (25), damped singular value decomposition (DSVD) algorithm based on SVD (26), and OMP algorithm based on matching pursuit (20)—these algorithms include regularization algorithm and greedy algorithm—was used to evaluate the performance of the framework combined with various reconstruction algorithms. In addition, to compare this CLT framework’s performance with those of other ROI methods, ISPR and TWD methods are introduced (21, 22). The parameters of all reconstruction algorithms and methods take their default values. The results prove that the P-EDDRS framework for CLT can combine various algorithms to reconstruct radioactive sources of different sizes and shapes, which has higher applicability. In addition, *in vivo* experiments show that the framework is still reliable and stable in living animals.

## 2 Method

### 2.1 Inverse Problem

Most of the energy of CR is concentrated in the short wavelength band, characterized by high scattering and low absorption in biological tissues. So the diffusion approximation (DA) model can be used as the mathematical basis to describe the Cerenkov photon transport process in tissues. DA models with Robin boundary conditions are often described as follows (27):


(1) $$\{\begin{array}{c} -\nabla \text{D}(r)\nabla \Phi (r)+\mu_{a}(r)\Phi (r)=\text{S}(r),r\in \Omega \\ \Phi (\xi )+2\text{Fnd}(\xi )=0,\xi \in \partial \Omega \\ \text{D}(r)=\frac{1}{3}\cdot {\left[(1-g)\cdot \mu_{s}+\mu_{a}\right]}^{-1}\\ \text{F}=\frac{1+{\text{R}}_{\text{f}}}{1-{\text{R}}_{\text{f}}} \end{array}$$

where ***Φ*** *(r)* denotes the Cerenkov photon flow rate at the point *r* in the region Ω, *μ_a_* and *μ_s_* are the absorption coefficient and scattering coefficient of a tissue, *g* is anisotropy factor, and ***D*** *(r)* denotes the diffusion coefficient at position *r*. The symbol ∇ is used for the differential operator of vector, 𝜕Ω is the set of the surface (boundary) points, and ξ is the point on the surface of a tissue. ***R*** *_f_* is the internal indicator of refraction of the tissue, and ***n*** is the unit normal vector whose direction is from the inside of the biological tissues to the outside of 𝜕Ω. Furthermore, the continuous space in biological tissues can be discretized into finite units by using the finite element method (FEM). By using FEM in solving Eq. (1), a reduced linear relationship between the unknown Cerenkov source distribution in the tissue and the surface photon flow rate can be obtained:


(2) $${\text{A}}_{\text{M}\times N}\times_{N\times 1}={\text{B}}_{M\times 1}$$

where ***A*** is the CLT system matrix, and it gives the mathematical process of Cerenkov photon propagation in the tissue, ***B*** represents the Cerenkov photon flow rate vector on the surface of biological tissues measured by a susceptible CCD camera, length *M* of ***B*** represents the semaphore measured, ***X*** represents the unknown Cerenkov source distribution vector in the organism, and length *N* of ***X*** represents number of grid nodes of FEM.

In essence, most of feasible region iterative reconstruction methods are essentially subtracted columns from ***A*** to simplify the process of solving Eq. (2). For example, the TWD method divides the initial reconstruction region into positive domain (POS), negative domain (NEG), and boundary domain (BND); combines POS and BND into ROI; and deletes the system matrix column corresponding to NEG. In the ISPR method, each iteration’s reconstruction results are arranged in descending order according to the energy intensity of nodes, and the columns of system matrix corresponding to nodes with lower energy are removed. Those iterative reconstruction methods based on feasible region have defects as mentioned in the *Introduction*. To optimize the feasible region method in CLT and improve the performance of traditional algorithms in CLT reconstruction, the P-EDDRS framework is proposed in this paper.

### 2.2 Probabilistic Energy Distribution Density Region Scaling Framework for Cerenkov Luminescence Tomography

The P-EDDRS framework for CLT mainly includes the following steps:

1. Reconstruct the CR source based on ROI (initial ROI is global).

2. Evaluate the quality of each iteration reconstruction result and generate probabilistic source distributions based on the evaluation.

3. According to the reconstruction source distribution density, determine the subsequent ROI.

4. Dynamically change the next ROI size based on the energy intensity of the current result.

5. Evaluate whether the cutoff conditions are met and continue if so; otherwise, return to step 1.

6. The results are post-processed and weighted to get the final Cerenkov source distribution when the iteration is complete.

**Figure 1:**
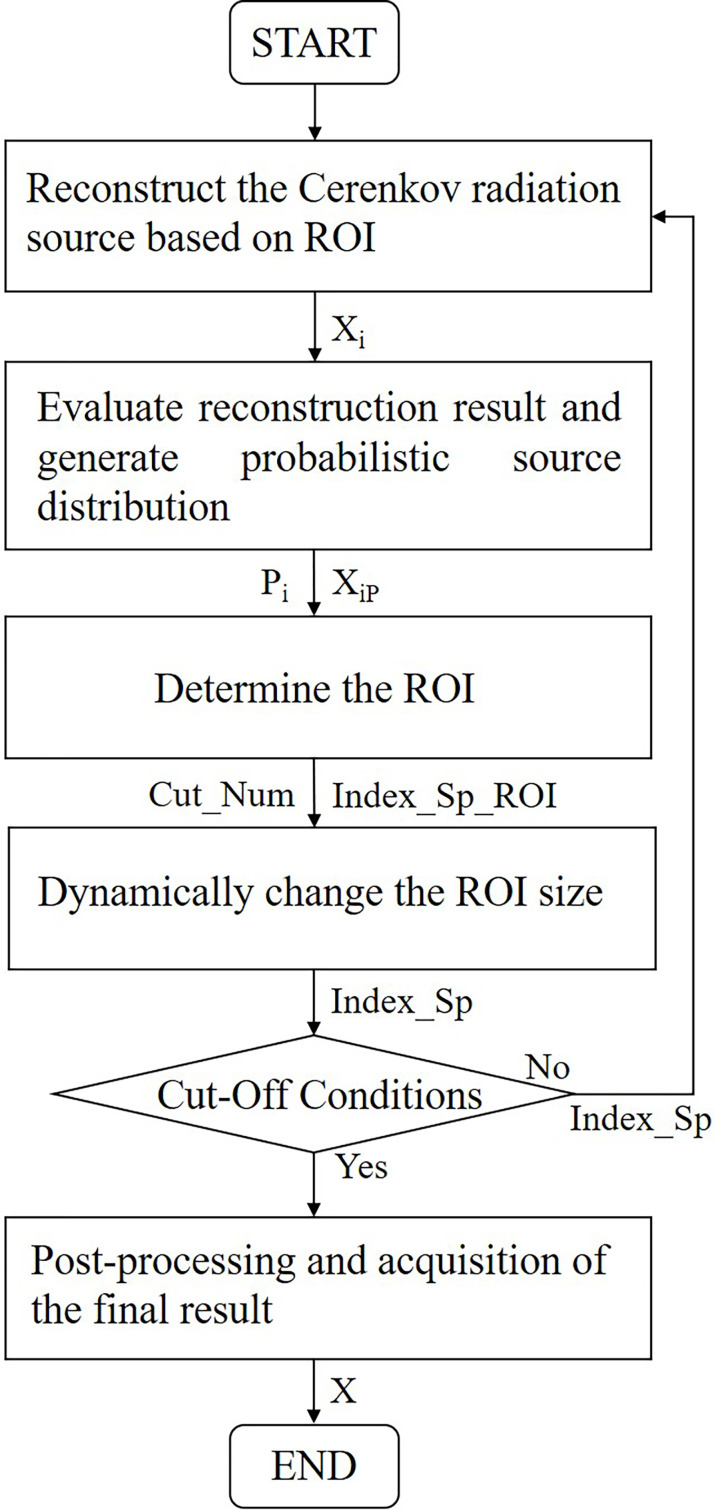
Flowchart of P-EDDRS framework for CLT. P-EDDRS, probabilistic energy distribution density region scaling; CLT, Cerenkov luminescence tomography.

**Figure 1** shows the flowchart of the P-EDDRS framework for CLT.

### 2.3 Evaluate Reconstruction Result and Generate Probabilistic Source Distribution

To evaluate the error of each CLT reconstruction radiation source distribution, this section performed the following actions.

First, the index named *Index_Sp* is initialized based on the global number of nodes. Next, according to *Index_Sp*, the reconstruction algorithm mentioned above is used to reconstruct the current region (the first time is the whole region) radiation source distribution, and the first iteration reconstruction source distribution ***X*** *_1_* is obtained. According to Eqs (3) and (4), the L_2_ norm error rate $E_{L2(x_{1})}$ and the cosine similarity $E_{Cos(x_{1})}$ of ***X*** *_1_* can be obtained, respectively.


(3) $$E_{L2({\text{X}}_{i})}={\left||{\text{A}}_{\left(,Index\_Sp\right)}\cdot {\text{X}}_{i}-\text{B}|\right|}_{2}$$

(4) $$E_{Cos({\text{X}}_{i})}=\frac{\Sigma [{\text{A}}_{\left(,Index\_Sp\right)}\cdot {\text{X}}_{i}\cdot \text{B}]}{{\left||A_{\left(,Index\_Sp\right)}\cdot {\text{X}}_{i}|\right|}_{2}\cdot {\left||\text{B}|\right|}_{2}}$$

where *i* is the number of iterations between 1 and the maximum number of iterations *L_max_* 50.

For now, evaluation of the reconstruction is obtained through Eqs (3) and (4). In order to assign the corresponding weight to each reconstruction source distribution in the overall framework, the weight value of each iteration reconstruction source distribution is introduced. The weight here essentially represents the probability value that the result of each iteration is the final result, so we can call this weight value as the probability weight value. Furthermore, from the reconstruction perspective, the L_2_ norm error is an inversely proportional evaluation. To achieve this effect, introduce Eq. (5).


(5) $$P_{L2({\text{X}}_{i})}=\frac{1./\Sigma_{j=1}^{i}E_{L2(X_{j})}}{\Sigma [1./\Sigma_{j=1}^{i}E_{L2(X_{j})}]}$$

The other indicator *E_cos_* is different from *E_L2_*. The closer the similarity is to 1, the more reliable the reconstruction source distribution is. It is a proportional relationship, which can be represented by Eq. (6).


(6) $$P_{Cos({\text{X}}_{i})}=\frac{\Sigma_{j=1}^{i}E_{L2(Xj)}}{\Sigma [\Sigma_{j=1}^{i}E_{L2(X_{j})}]}$$

Now, two metrics can use to evaluate each outcome. To integrate these two indexes, Eq. (7) can be introduced. Eq. (7) gives the result of each reconstruction source distribution corresponding probability weight value. When all iterations are completed, each iteration’s initial reconstruction source distribution is multiplied by the corresponding probability weight, and the sum is the final reconstruction source distribution.


(7) $$P_{Err({\text{X}}_{i})}=\frac{P_{L2(X_{i})}+P_{Cos(X_{i})}}{2}$$

Therefore, the normalized probabilistic result of each reconstruction source distribution can be expressed by Eq. (8). Through Eq. (8), the initial reconstruction source distribution is transformed into probabilistic reconstruction source distribution with weights. The distribution can be combined with the corresponding grid coordinates as a group of random variables distributed in 3D space to determine the ROI region in the next section.


(8) $${\text{X}}_{iP}=\frac{{\text{X}}_{i}\cdot P_{Err({\text{X}}_{i})}}{\Sigma {\text{X}}_{i}\cdot P_{Err(X_{i})}}$$

### 2.4 Determine the Region of Interest

After the probabilistic source distribution of one reconstruction in *Section 2.3* is obtained, the feasible area of the subsequent reconstruction needs to be determined. We assume that the probabilistic source distribution ***X*** *_iP_* is a set of random variables whose mathematical expectation is *μ* and variance is σ^2^. For any positive number *K*, Chebyshev’s inequality holds as in Eq. (9).


(9) $$P\left\{\left|{\text{X}}_{iP}-\mu \right|\;K\right\}\geq 1-\frac{\sigma^{2}}{K^{2}}$$

Further, *K* is expressed as different values:


(10) $$\{\begin{array}{l} P\left\{\left|{\text{X}}_{iP}-\mu \right|\;2\sigma \right\}\geq 1-\frac{1}{4}\\ P\left\{\left|{\text{X}}_{iP}-\mu \right|\;4\sigma \right\}\geq 1-\frac{1}{16} \end{array}$$

It can be seen from the above formula that the distribution of random variables has its inherent trend. According to this feature, assume that all nodes with corresponding grid coordinates are random variables distributed in 3D space. The distribution of random variables in 3D space also has the rule of Eq. (10). In other words, the distribution of all reconstruction source distribution tends to be close to its mathematical expectation, which is the basis for determining ROI. According to the characteristics of Eq. (10), cuboid can be used as the form of ROI. Now, we have a center point, the length of the sides in the three directions and the deflection angle concerning the coordinate axes are determined, and the form of ROI (cuboid) in space can be determined. The average probability of nodes can be obtained by Eq. (11) as the center of the ROI region.


(11) $$\{\begin{array}{l} \bar{x_{P}}=\Sigma_{t=1}^{N}x_{t}\cdot {\text{x}}_{iP(t)}\\ \bar{y_{P}}=\Sigma_{t=1}^{N}y_{t}\cdot {\text{x}}_{iP(t)}\\ \bar{z_{P}}=\Sigma_{t=1}^{N}z_{t}\cdot {\text{x}}_{iP(t)} \end{array}$$

where *N* represents the total number of nodes in the current iteration; *x_t_*, *y_t_*, and *z_t_* represent the coordinates of the point *t*; ***x*** *_iP(t)_* is the *t*th element in ***X*** *_iP_*; $\bar{x_{P}}$ , $\bar{y_{P}}$ , and $\bar{z_{P}}$ represent the probabilistic average value of the corresponding coordinate.

Once the center is determined, the deflection angle and side length of ROI need to be determined. The covariance matrix *M_cov_* is introduced to solve the side length and deflection angle of ROI.


(12) $$M_{\mathrm{cov}}=\left[\begin{array}{lll} \sigma_{x}^{2} & Cov_{xy} & Cov_{xz}\\ Cov_{xy} & \sigma_{y}^{2} & Cov_{yz}\\ Cov_{xz} & Cov_{yz} & \sigma_{z}^{2} \end{array}\right]$$

where $\sigma_{x}^{2},\sigma_{y}^{2},and\;\sigma_{z}^{2}$ represent the probability variance of each coordinate direction; and *Cov_xy_*, *Cov_yz_* , and *Cov_xz_* represent the covariance between different coordinate directions. These two variables are represented by Eqs (13) and Eq. (14):


(13) $$\{\begin{array}{l} \sigma_{x}^{2}=\Sigma_{t=1}^{N}[{\left(x_{t}-\bar{x_{P}}\right)}^{2}\cdot {\text{x}}_{iP(t)}]\cdot \frac{N}{N-1}\\ \sigma_{y}^{2}=\Sigma_{t=1}^{N}[{\left(y_{t}-\bar{y_{P}}\right)}^{2}\cdot {\text{x}}_{iP(t)}]\cdot \frac{N}{N-1}\\ \sigma_{z}^{2}=\Sigma_{t=1}^{N}[{\left(z_{t}-\bar{z_{P}}\right)}^{2}\cdot {\text{x}}_{iP(t)}]\cdot \frac{N}{N-1} \end{array}$$

(14) $$\{\begin{array}{l} Cov_{xy}=\Sigma_{t=1}^{N}(x_{t}-\bar{x_{P}})\cdot (y_{t}-\bar{y_{P}})\cdot {\text{x}}_{iP(t)}\cdot \frac{N}{N-1}\\ Cov_{yz}=\Sigma_{t=1}^{N}(y_{t}-\bar{y_{P}})\cdot (z_{t}-\bar{z_{P}})\cdot {\text{x}}_{iP(t)}\cdot \frac{N}{N-1}\\ Cov_{xz}=\Sigma_{t=1}^{N}(x_{t}-\bar{x_{P}})\cdot (z_{t}-\bar{z_{P}})\cdot {\text{x}}_{iP(t)}\cdot \frac{N}{N-1} \end{array}$$

All parameters are multiplied by *N*/(*N* − 1) to ensure unbiased estimates of the variance and covariance. Through the above calculation, the construction of the overall probability result covariance matrix is completed. Further, the eigenvalues ***Val*** and eigenvectors ***Vec*** of the matrix can be obtained. ***Val*** and ***Vec*** are in the form of matrices, in which the three diagonal elements of ***Val*** represent the corresponding eigenvalues in the three coordinate directions. At the same time, the ***Vec*** is the eigenvector in the three coordinate directions. To realize dynamic scaling of the region, the ROI side length *R* can be defined as in Eq. (15):


(15) $$R=\left[\sqrt{\left|Val_{\left(1,1\right)}\right|,}\sqrt{\left|Val_{\left(2,2\right)}\right|},\sqrt{\left|Val_{\left(3,3\right)}\right|,}\right]\cdot Size$$

where *Size* is the coefficient used to control the ROI, which is initially set to 1 and will be refreshed in *Section 2.5*.

By Eqs (11)–(15), a center $\left(\bar{x_{P}},\bar{y_{P}},\bar{z_{P}}\right)$ , three directions (***Vec*)**, and the corresponding length of the sides (***Val***) can be acquired. Assuming Eq. (14) to get to the center of the origin, the ROIs of the cube eight vertices are (+, + +), (+, −, +), (−, −, +), (+, −, +), (+, −, −), (−, −, −), (+, −, −), and (+, +, −). After the positive and negative signs are determined, the vector direction from the origin to each vertex can be deflected by multiplying the corresponding feature vectors. The center point obtained by Eq. (11) can be added to obtain the eight vertices of the current ROI. At the same time, we can dynamically adjust the scaling rate of next ROI by multiplying the size variable introduced in *Section 2.5* with ***Val***.

When the ROI is determined, nodes among this ROI can be detected, and calculate the spatial distance between the nodes in the ROI region and the ROI center and arrange them in ascending order to get the ROI node index named *Index_Sp_ROI*.

**Figure 2:**
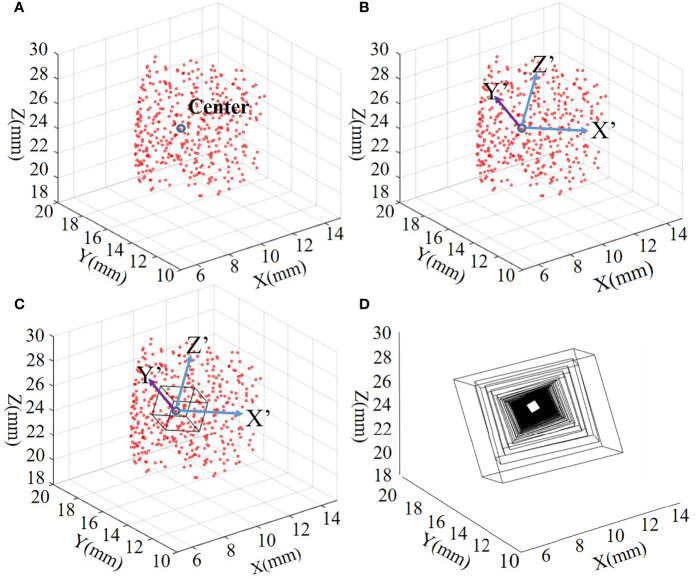
**(A–C)** Examples of one of the ROI division processes. **(D)** The ROI trajectory of the entire iterative process. ROI, region of interest.

**Figure 2** shows how the ROI is generated. **Figures 2A–C** show examples of one of the ROI division processes, and **Figure 2D** shows examples of all ROI tracks of the overall process.

### 2.5 Dynamically Change the Region of Interest Size

The previous step identified the ROI area, but the ROI rate of change will be significantly accelerated during the mid to late iteration phases. This phenomenon will cause some nodes to be directly classified outside the ROI, which will lead to the loss of the morphological information of the radiation source. To better retain the nodes that may be the radiation source and more information about the energy of radiation source, the change rate of ROI should be controlled. So this paper introduces the formula of Naser et al., as follows (23):


(16) $$\beta ={\left(\frac{Cut\_Num}{Num_{f}}\right)}^{\frac{1}{L_{\max}-1}}$$

where *Num_f_* is the expected number of nodes remaining in the final iteration, because the grid is used as the division unit of spatial structure, and this value is set as 4 (a tetrahedron). *Cut_Num* is size of *Index_Sp_ROI* at first iteration, and *ß* represents the attenuation coefficient.

Now, a judgment condition between the size relationship between *Cut_Num/ß* ^2^ and the *Cut_Num* has been set: if the quotient is greater than 2, the *Size* in Eq. (15) is set to 2; if it is less than 1, the *Size* is set to 0.5; in other cases, the *Size* is set to 1. This variable enables the ROI to shrink or expand.

After the change of ROI region is completed, the current *Index_Sp* is arranged in descending order according to the energy intensity value of the CLT reconstruction source distribution. The new *Cut_Num* is made by dividing the current *Cut_Num* by *ß* and rounding the number toward positive.

The previous *Cut_Num* nodes of *Index_Sp* are taken as the index of the current threshold shrinking to generate *Index_Sp_Descend*.

Concatenate index *Index_Sp_Descend* after *Index_Sp_ROI*, and a new index is obtained, which contains the nodes of the ROI region and the nodes with the threshold shrunk. After the duplicate elements of this new index are removed, use *Cut_Num* to cut off the new index, and the remaining elements are the *Index_Sp*, which will be used for the following CLT reconstruction. When the remaining elements are still greater than 1 and the number of iterations does not reach *L_max_*, return to *Section 2.3*.

### 2.6 Post-Processing and Acquisition of the Final Result

At the end of the overall iteration, all iteration reconstruction results to ***X*** *_i_*, and the corresponding probability error rate *P_Err(_* ***_X_***_i_ *_)_* has been obtained. Although all reconstruction source distribution has been recorded, some iterations have significantly higher probabilistic error rates. From the description by Ding et al., the iteration result error evaluation of 3D reconstruction tends to be in the form of Gaussian distribution (13). Here, the error evaluation can be used as the criterion for evaluating the results, so the mean value and standard deviation for *E_L2_*_(_ ***_X_*** *_i)_* and *E_cos(_* ***_X_*** *_i)_* can be calculated. According to the definition of Gaussian distribution, only the iteration source distribution that is centered on the mean and within one times the standard deviation is retained. The intersection of the two Gaussian filtering results is the result of some iterations that need to retain. Once this step is complete, use Eqs (5)–(7) again to generate the new parameters and *P’_Err(_* ***_X_***_i_ *_)_*. The final CLT reconstruction source distribution ***X*** *_final_* can be obtained by Eq. (17).


(17) $${\text{X}}_{final}=\Sigma_{i=1}^{K}P\prime_{Err(X_{i})}\cdot {\text{X}}_{i}$$

where *K* is the number of remaining results after the completion of filtering treatment in this section.

## 3 Experiments and Results

The CLT reconstruction experiments were performed in MATLAB 2020B and run on a desktop computer with a 3.00-GHz Intel Core i5 CPU and 32 GB of memory.

To verify and systematically evaluate the performance and characteristics of the CLT reconstruction framework proposed in this paper, several groups of CLT numerical simulation experiments and CLT *in vivo* experiments were designed. The location error (*E_L_*), the dice coefficient (*Dice*), the tetrahedral volume ratio (*R_V_*), and the global relative residual (*R_R_*) are introduced as quantitative evaluation indicators.

The location error *E_L_* is defined as the Euclidean distance between the reconstruction source distribution center point coordinates *(x,y,z)* and the actual radiation source coordinates (*x_0’_y_0’_z_0_*):


(18) $$E_{L}=\sqrt{{\left(x-x_{0}\right)}^{2}+{\left(y-y_{0}\right)}^{2}+{\left(z-z_{0}\right)}^{2}}$$

Dice coefficient is used to evaluate the degree of shape similarity (overlap) between the reconstructed source distribution area *R* and the actual radiation source distribution area *T*. The closer it is to 1, the higher the similarity between *R* and *T* is. The following formula defines Dice coefficient:


(19) $$Dice=2\frac{\left|R\cap T\right|}{\left|R\right|+\left|T\right|}$$

The tetrahedral volume ratio *R_V_* is defined as the ratio of the tetrahedral volume *V_T_* of the actual radiation source distribution to the tetrahedral volume *V_R_* of the reconstruction source distribution. Same as *Dice*, when *R_V_* is closer to 1, the higher the size similarity of the radiation source. The following formula calculates *R_V_*:


(20) $$R_{V}=\frac{V_{T}}{V_{R}}$$

The final theoretical error of the evaluation result of global relative residual *R_R_* is calculated by the following equation: the lesser the *R_R_*, the smaller the theoretical error.


(21) $$R_{R}=\frac{{\left||\text{B}-\text{A}\cdot {\text{X}}_{final}|\right|}_{2}}{{\left||\text{B}|\right|}_{2}}$$

### 3.1 Numerical Simulations

**Figure 3:**
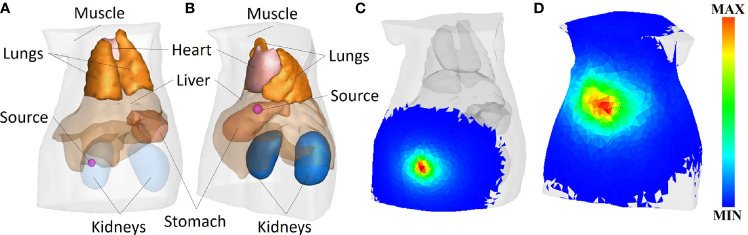
**(A)** Model of spherical radiation source radius of 0.8 mm at (9.5, 15.5, 25) mm. **(B)** Model of spherical radiation sources radius of 1.25 mm at (20, 8, 15) mm. **(C, D)** The surface photon energy distributions corresponding to panels **(A, B)**, respectively.

In this section, a non-homogeneous digital mouse model show in **Figure 3** is applied to verify the performance of the CLT reconstruction framework (28). To reduce the computational complexity and performance cost, the head and tail of the digital rat were removed, and only the trunk containing the main organs was retained, which was composed of the heart, lung, liver, stomach, and kidney, while the rest were muscle tissues. 2-[^18^F]-Fluoro-2-deoxy-d-glucose (^18^F-FDG) was the Cerenkov radioactive source used. In the CLT numerical simulation, Cerenkov photons generated by ^18^F radioactive source in tissues were simulated by GEANT4, and the transmission process of these Cerenkov photons in tissues was simulated by MOSE (29–32).

The CR signal with wavelength of 630 nm was collected for reconstruction. The optical parameters of each organ and tissue are given in reference (33), as shown in **Table 1**. In the CLT numerical simulation experiment, the radiation source is distributed in two positions, namely, (9.5, 15.5, 25) mm and (20, 8, 15) mm, using different shapes and sizes. One set of shapes is shown in **Figures 3A–D** show the surface Cerenkov photon energy distribution maps corresponding to **Figures 3A, B**.

**Table 1:** Optical parameters of different tissues and organs of the numerical mouse.

Material	*μ_α_*/mm^-1^	*μ_s’_*/mm^-1^	*g*
Muscle	0.016	0.510	0.9
Heart	0.011	1.053	0.86
Stomach	0.002	1.525	0.9
Liver	0.065	0.723	0.9
Kidney	0.012	2.472	0.9
Lung	0.036	2.246	0.9

Four groups of experiments were designed in this section. The first group of experiments uses Tikhonov, DSVD, LassoLSQR, and OMP algorithms mentioned above to reverse reconstruct the radiation source in combination with the framework to verify the feasibility of this framework. The second set of experiments compared this framework with ISPR and TWD iterative reconstruction methods to verify the framework’s effectiveness in CLT. In the third and fourth groups, radiation sources of different sizes and shapes were selected for reconstruction to verify the stability and robustness of the framework in CLT.

#### 3.1.1 The Experiment of Feasibility Verification

**Figure 4:**
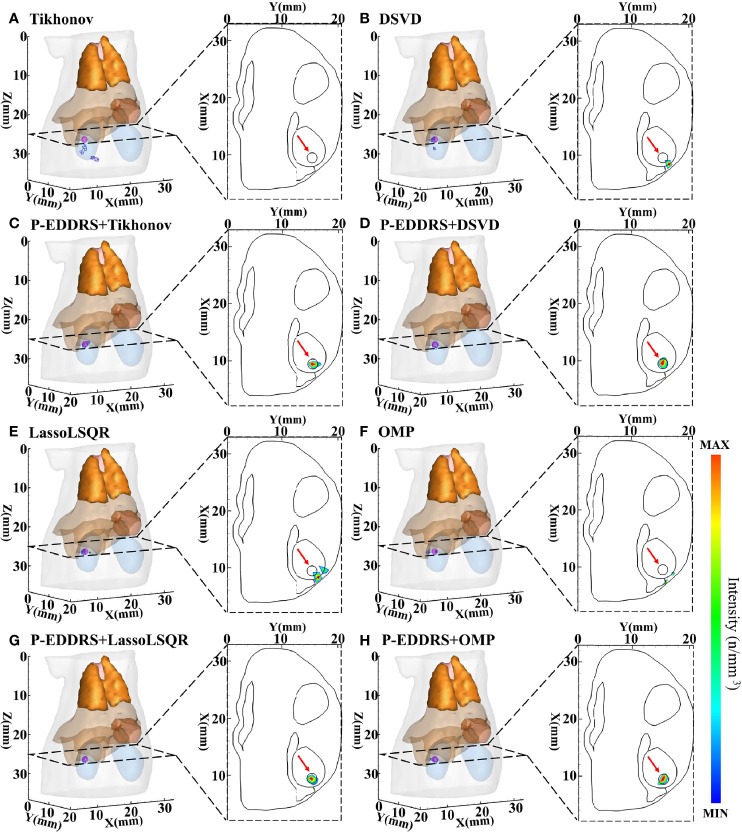
Results of P-EDDRS framework feasibility verification in CLT. All results include 3D views and corresponding cross-section views at the plane of Z = 25 mm. **(A, B, E, F)** The initial reconstruction source distribution of Tikhonov, DSVD, LassoLSQR, and OMP algorithms, respectively. **(C, D, G, H)** The radiation source distribution obtained by the corresponding algorithms of panels **(A, B, E, F)** after using P-EDDRS framework. P-EDDRS, probabilistic energy distribution density region scaling; CLT, Cerenkov luminescence tomography; DSVD, damped singular value decomposition; LassoLSQR, Lasso and Least Square QR-factorization; OMP, orthogonal matching pursuit.

This experiment was designed to verify the feasibility of the proposed framework in CLT and the versatility of different algorithms. The digital mouse has been discretized into a grid of 12,831 nodes and 66,085 tetrahedrons using Amira (Visage Imaging, Australia). A spherical radiation source with a radius of 0.8 mm was placed at coordinates (9.5, 15.5, 25) mm as shown in **Figure 3A**. The results are shown in **Figure 4**, and the quantitative indexes are shown in **Table 2**.

**Table 2:** Quantitative evaluation results of P-EDDRS framework feasibility verification in CLT.

Method	Reconstructed center/mm	*E_L_*/mm	*Dice*	*R_V_*	*R_R_*
Tikhonov	9.11, 15.62, 27.67	2.71	0.11	0.64	0.828
P-EDDRS+Tikhonov	9.60, 15.92, 24.71	0.52	0.72	0.88	0.694
DSVD	8.34, 16.63, 24.86	1.62	0.12	1.50	0.846
P-EDDRS+DSVD	9.80, 15.48, 25.21	0.37	0.87	0.82	0.757
LassoLSQR	7.22, 16.06, 24.74	2.36	0	1.32	0.860
P-EDDRS+LassoLSQR	9.85, 15.51, 25.25	0.43	0.87	0.82	0.859
OMP	8.34, 16.63, 24.86	1.62	0.12	1.39	0.880
P-EDDRS+OMP	9.50, 15.28, 24.96	0.22	0.86	1.14	0.847

P-EDDRS, probabilistic energy distribution density region scaling; CLT, Cerenkov luminescence tomography; DSVD, damped singular value decomposition; LassoLSQR, Lasso and Least Square QR-factorization; OMP, orthogonal matching pursuit.

In **Figure 4**, the radiation source is marked in red, and the reconstruction source distribution is shown in a purple grid in all 3D views. In all axial views, the actual location of the radiation source is marked by a red arrow. It can be seen that in all four algorithms, the reconstruction source distribution is significantly improved after the framework is used, and different algorithms can realize convergence after several iterations and obtain better reconstruction results. Specifically, Tikhonov and DSVD algorithms have poor convergence in one-step reconstruction results and cannot determine the actual radiation source position. With P-EDDRS, the correct radiation source location can be obtained. LassoLSQR and OMP algorithms’ initial result is incorrect and difficult to converge into a single region. After using the framework, the reconstruction results can be corrected. In addition, in terms of the degree of shape recovery, this framework can better restore the shape of the radiation source, which can be proved from the pictures and the Dice coefficient. It can also be seen from RR in **Table 2** that the results of this framework are closer to the theoretically more correct results.

#### 3.1.2 The Experiment of Efficiency Verification

**Figure 5:**
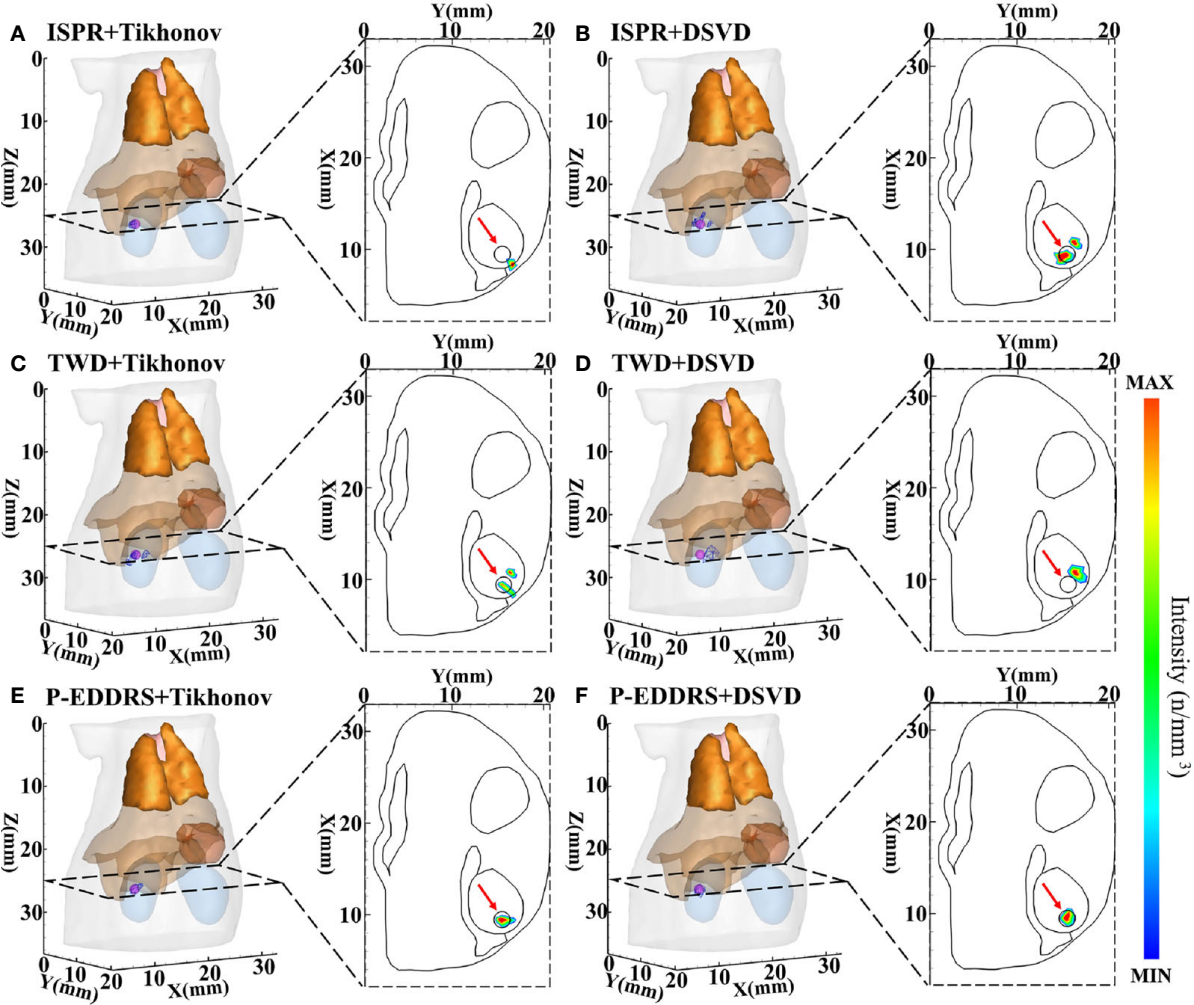
Results of P-EDDRS framework efficiency verification in CLT. All results include 3D views and corresponding cross-section views at the plane of Z = 25 mm. **(A, B)** The initial reconstruction source distribution of Tikhonov and DSVD algorithms in ISPR method, respectively. **(C, D)** The reconstruction source distribution of Tikhonov and DSVD algorithms in TWD strategy, respectively. **(E, F)** The reconstruction source distribution of Tikhonov and DSVD algorithms in our framework, respectively. P-EDDRS, probabilistic energy distribution density region scaling; CLT, Cerenkov luminescence tomography; DSVD, damped singular value decomposition; ISPR, iterative shrinking permissible region; TWD, three-way decision.

This experiment was designed to compare the performance of the proposed framework with two other typical ROI shrinking methods: TWD and ISPR (21, 22) in CLT reconstruction. The grid used and the radiation source placement are the same as those of the last experiment. The Tikhonov and DSVD algorithms mentioned above were used to reverse reconstruct the radiation source combined with the framework. The results are shown in **Figure 5**, and the quantitative indexes are shown in **Table 3**.

**Table 3:** Quantitative evaluation results of P-EDDRS framework efficiency verification in CLT.

Method	Reconstructed center/mm	*E_L_*/mm	*Dice*	*R_V_*	*R_R_*
ISPR+Tikhonov	8.34, 16.63, 24.86	1.62	0.15	2.37	0.891
ISPR+DSVD	9.03, 15.10, 24.77	0.66	0.44	0.39	0.980
TWD+Tikhonov	8.95, 15.92, 24.91	0.70	0.49	0.46	0.656
TWD+DSVD	10.81, 16.17, 24.96	1.47	0.16	1.98	0.923
P-EDDRS+Tikhonov	9.60, 15.92, 24.71	0.52	0.72	0.88	0.604
P-EDDRS+DSVD	9.80, 15.48, 25.21	0.37	0.87	0.82	0.757

P-EDDRS, probabilistic energy distribution density region scaling; CLT, Cerenkov luminescence tomography; ISPR, iterative shrinking permissible region; DSVD, damped singular value decomposition; TWD, three-way decision.

In **Figure 5**, the radiation source is marked in red, and the reconstruction source distribution is shown in a purple grid in all 3D views. In all axial views, the actual location of the radiation source is marked by a red arrow. As shown in **Figure 5A**, ISPR leads Tikhonov algorithm results to tend to the point with high energy intensity value, and the correct radiation source depth cannot be obtained. At the same time, in **Figure 5B**, similar trends tend to be of high energy intensity but ignore the shape. Because ISPR determines the ROI region based on node energy drop ranking, spatial information between nodes is ignored, resulting in the follow-up’s discontinuous morphological distribution, leading to multiple high energy points. The value of *R_R_* under TWD+Tikhonov condition is smaller than that under P-EDDRS+DSVD condition. However, the latter result is more accurate whether evaluated from other quantitative indicators, 3D views, or cross-section views. As shown in **Figures 5C, D**, TWD can obtain the actual radiation source depth relatively closely. However, the results of TWD depend entirely on the previous iteration, which will lead to the accumulation of error results and finally get the error distribution. In **Figures 5E, F**, our framework can achieve better results among the three methods in terms of the results of the two algorithms, whether it is positioning error, Dice coefficient representing morphology, global relative residual, or the volume of reconstructed radiation source distribution. In addition, the depth and distribution of the radiation source are closer to the actual location.

#### 3.1.3 The Stability and Robustness Experiment I

**Figure 6:**
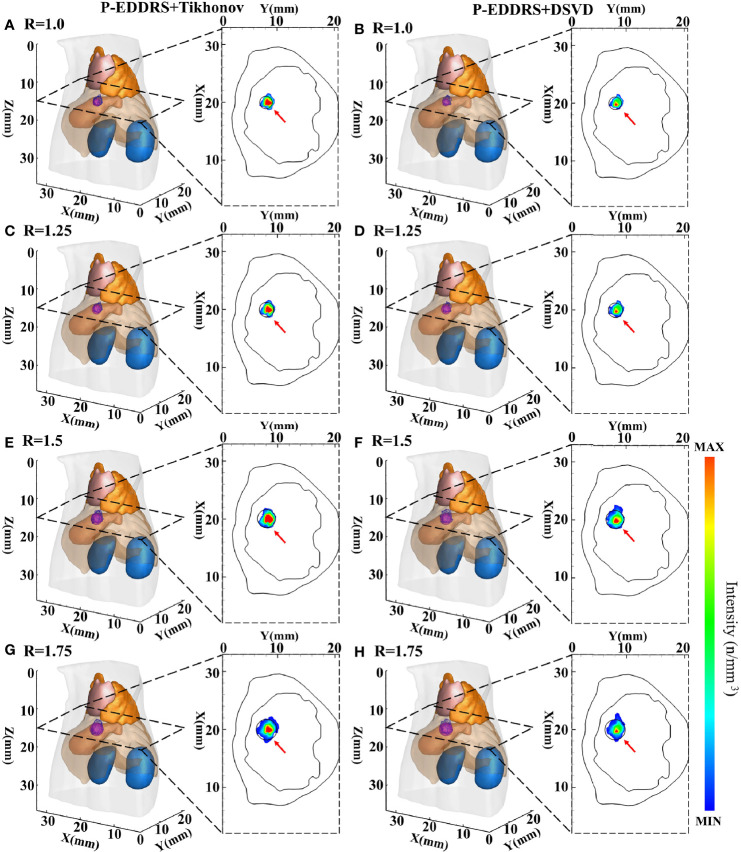
Results of P-EDDRS framework stability and robustness experiment I. All results include 3D views and corresponding cross-section views at the plane of Z = 15 mm **(A, C, E, G)**. The reconstruction source distribution of spheres with radii of 1.0, 1.25, 1.5, and 1.75 mm combined with Tikhonov algorithm in our framework, respectively **(B, D, F, H)**. The reconstruction source distribution of spheres with radii of 1.0, 1.25, 1.5, and 1.75 mm combined with DSVD algorithm in our framework, respectively. P-EDDRS, probabilistic energy distribution density region scaling; DSVD, damped singular value decomposition.

This experiment is to verify the stability and robustness of the P-EDDRS framework for different radiation source sizes in CLT. Four spherical radiation sources with different radii and sizes are used as targets. The digital mouse has been discretized into a grid of 28,463 nodes and 159,957 tetrahedrons in the same way. The spherical radiation source was placed at coordinates (20, 8, 15) mm as shown in **Figure 3B**. Different from the first two, four radiation sources of different sizes have been placed at grid in this experiment, with radii of 1.0, 1.25, 1.5, and 1.75 mm. The Tikhonov and DSVD algorithms mentioned above were used to reverse reconstruct the radiation source in combination with the framework. The results are shown in **Figure 6**, and the quantitative indexes are shown in **Table 4**.

**Table 4:** Quantitative evaluation results of P-EDDRS framework stability and robustness experiment I.

Method	Size/mm	Reconstructed center/mm	*E_L_*/mm	*Dice*	*R_V_*
P-EDDRS+Tikhonov	R = 1.00	19.92, 8.38, 14.68	0.50	0.71	0.82
P-EDDRS+DSVD	R = 1.00	19.65, 8.51, 14.57	0.76	0.72	0.87
P-EDDRS+Tikhonov	R = 1.25	19.92, 8.38, 14.68	0.50	0.80	1.23
P-EDDRS+DSVD	R = 1.25	19.65, 8.50, 14.56	0.75	0.73	1.15
P-EDDRS+Tikhonov	R = 1.50	19.92, 8.38, 14.68	0.50	0.78	1.24
P-EDDRS+DSVD	R = 1.50	19.64, 8.51, 14.55	0.77	0.80	0.95
P-EDDRS+Tikhonov	R = 1.75	19.92, 8.38, 14.68	0.50	0.78	1.09
P-EDDRS+DSVD	R = 1.75	19.64, 8.50, 14.57	0.75	0.76	0.97

P-EDDRS, probabilistic energy distribution density region scaling; DSVD, damped singular value decomposition.

In **Figure 6**, the radiation source is marked in red, and the reconstruction source distribution is shown in a purple grid in all 3D views. In all axial views, the actual location of the radiation source is marked by a red arrow. As shown from the results in **Figure 6**, our framework can achieve better performance in four different sizes of experiments, and all the indicators are satisfactory. From all the results, the *E_L_* of all results is between 0.50 and 0.77, and the Dice coefficient remained above 71%. All but the minor radiation source is close to the actual volume. Because the finite element grid method is adopted in this paper, it has a specific size. If the radiation source is too small, the grid cannot fit the radiation source volume well. This phenomenon leads to some errors in the reconstruction of small size radiation sources. This is the limitation of the grid reconstruction method. This experiment proves that different reconstruction algorithms can estimate the size of radiation sources effectively under this framework in CLT.

#### 3.1.4 The Stability and Robustness Experiment II

**Figure 7:**
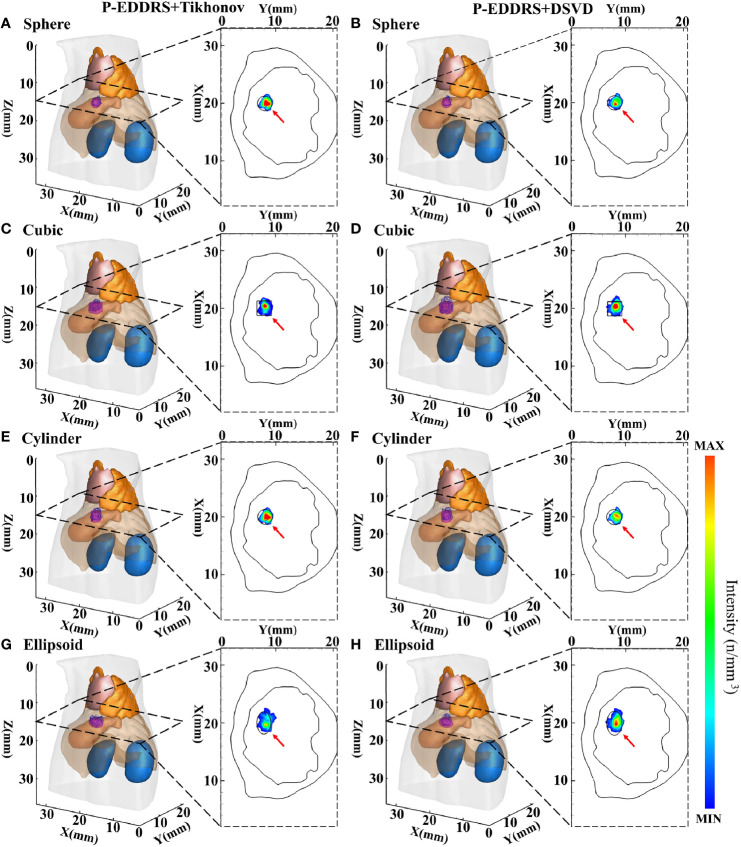
Results of P-EDDRS framework stability and robustness experiment II. All results include 3D views and corresponding cross-section views at the plane of Z = 15 mm **(A, C, E, G)**. The reconstruction source distribution of different shapes with sphere, cubic, cylinder, and ellipsoid combined with Tikhonov algorithm in our framework, respectively **(B, D, F, H)**. The reconstruction source distribution of different shapes with sphere, cubic, cylinder, and ellipsoid combined with DSVD algorithm in our framework, respectively. P-EDDRS, probabilistic energy distribution density region scaling; DSVD, damped singular value decomposition.

This experiment is similar to the last in verifying the stability and robustness of the P-EDDRS framework for different radiation source shapes in CLT. Four different shapes of radiation sources were placed in the grid: a sphere with a radius of 1.25, a cube with sides of 2.5, a cylinder with a radius of 1.25 and a height of 2.5, and an ellipsoid with a = 2 and b = c = 1.25. The grid is the same as in the last experiment. The radiation source was placed at coordinates (20, 8, 15) mm. The results are shown in **Figure 7**, and the quantitative indexes are shown in **Table 5**.

**Table 5:** Quantitative evaluation results of P-EDDRS framework stability and robustness experiment II.

Method	Shape	Reconstructed center/mm	*E_L_*/mm	*Dice*	*R_V_*
P-EDDRS+Tikhonov	Sphere	19.92, 8.38, 14.68	0.50	0.80	1.23
P-EDDRS+DSVD	Sphere	19.65, 8.50, 14.56	0.75	0.73	1.15
P-EDDRS+Tikhonov	Cubic	20.59, 8.27, 14.60	0.76	0.73	1.31
P-EDDRS+DSVD	Cubic	20.15, 8.13, 14.65	0.40	0.73	1.19
P-EDDRS+Tikhonov	Cylinder	19.92, 8.38, 14.68	0.51	0.80	1.15
P-EDDRS+DSVD	Cylinder	19.68, 8.54, 14.47	0.82	0.76	1.13
P-EDDRS+Tikhonov	Ellipsoid	19.45, 8.50, 14.65	0.82	0.65	1.08
P-EDDRS+DSVD	Ellipsoid	20.61, 8.52, 14.67	0.86	0.73	1.03

P-EDDRS, probabilistic energy distribution density region scaling; DSVD, damped singular value decomposition.

In **Figure 7**, the radiation source is marked in red, and the reconstruction source distribution is shown in a purple grid in all 3D views. In all axial views, the actual location of the radiation source is marked by a red arrow. From all the results, the *E_L_* of all results is between 0.50 and 0.86, and the Dice coefficient remained above 65%. All of the reconstruction source distribution volume is close to the actual radiation source volume. This experiment proves that different reconstruction algorithms can estimate the shape of radiation sources effectively under this framework in CLT.

### 3.2 *In Vivo* Experiments

**Figure 8:**
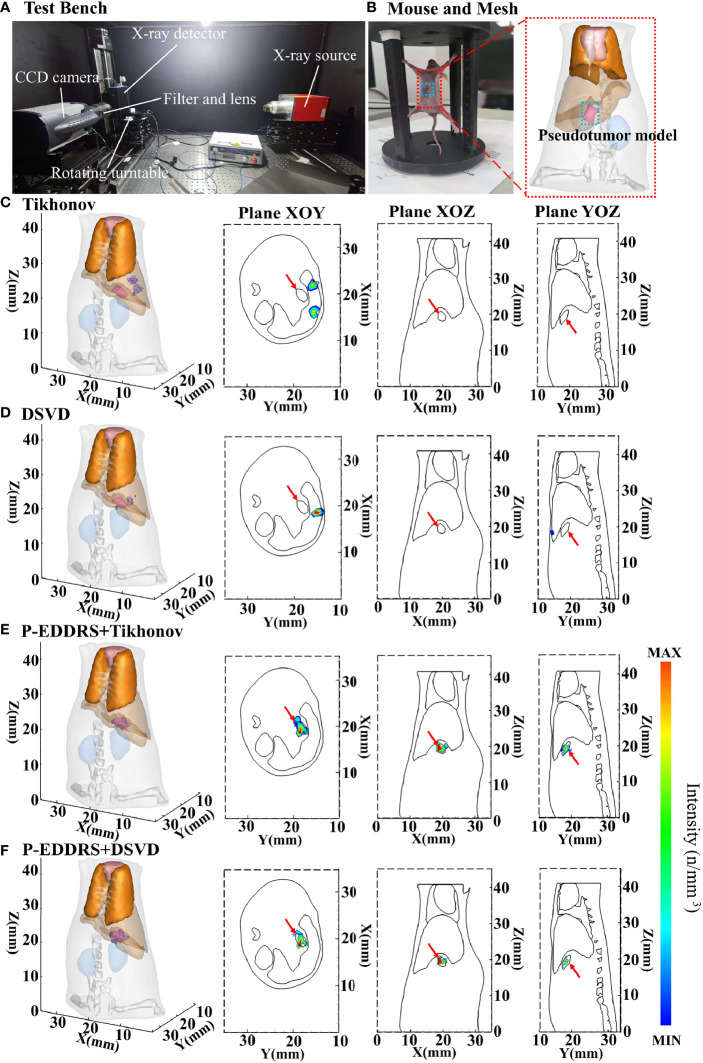
Results of the CLT *in vivo* pseudotumor experiments. All results include 3D views and corresponding cross-section views at the Z = 19.2 mm, Y = 18.4 mm, and X = 19.68 mm. **(A)** Picture of the test bench where the data were collected. **(B)** The mouse used, and the dashed box is the pseudotumor model planting area. **(C, D)** The initial reconstruction source distribution of Tikhonov and DSVD algorithms, respectively. **(E, F)** The reconstruction source distribution obtained by the corresponding algorithms of panels **(C, D)** after using our framework. CLT, Cerenkov luminescence tomography; DSVD, damped singular value decomposition.

To verify the feasibility and performance of our framework in an actual CLT situation, two groups of *in vivo* experiments are designed. The first group verifies the reconstruction ability of the framework in the case of relatively deep radioactive sources by using the pseudotumor model, and the second group verifies the reconstruction ability of the framework in the case of shallow radioactive sources by using subcutaneous breast cancer. Two approximately 7-week-old female nude mice (BALB/c Nude) served as the imaging model. All animal procedures were performed under isoflurane gas anesthesia (2% isoflurane–air mixture) to minimize the suffering of the mice. Animal experiments comply with the Regulations on the Management of Experimental Animals. All procedures follow the Animal Ethics Committee of the Northwest University of China (No. NWU-AWC-20210901M). The optical data were acquired using the iXon Ultra electron double CCD camera manufactured by ANDOR (Northern Ireland). The X-ray source is the L9181-02 microfocus ray source, and the X-ray detector is C7942CA-22, all manufactured by HAMAMATSU (Japan). The optical lens is EF 24 mm f/1.4L II USM manufactured by Canon (Japan). The band-pass filter is FF01-630/92-25 manufactured by Semrock (USA). The CCD camera is pre-cooled to −80°C, and the fuzzy local information C-means and curvature-driven diffusion (FLICMCDD) method in reference (34) is used to reduce the influence of noise signals. Exposure time is set to 5 min, the gain value is set to 300, shift speed is set to 13 μs, and the read rate is set to 1 MHz at 16 bits. In *in vivo* experiments, ^18^F-FDG is used as the Cerenkov radioactive source. The data acquisition equipment is shown in **Figure 8A**.

#### 3.2.1 The Experiment of Pseudotumor

The pseudotumor model used in this study was made of plastic and was a cylindrical tube with the most extended length of 2.3 mm, the widest width of 1.1 mm, and the highest length of 1.5 mm, as shown in **Figure 8B**. This pseudotumor model was injected with about 800 ± 50 μCi of ^18^F-FDG as Cerenkov radioactive source. The pseudotumor model was implanted in the mouse abdominal cavity, at the back of the left inner lobe of the liver, close to the liver, and implantation depth is deeper. The mouse was dispersed into a grid of 13,475 nodes and 69,923 tetrahedrons using FEM as shown in **Figure 8B**. This grid also removed the head and tail to reduce computational complexity, leaving only the main organs in the trunk. The difference from the numerical simulation is that this grid contains bones. The corresponding optical parameters of animal tissues were the same with numerical simulations. In this experiment, the approximate central coordinates of the pseudotumor model are (19.68, 18.40, 19.20) mm. The Tikhonov and DSVD algorithms mentioned above were used to reverse reconstruct it in combination with the P-EDDRS framework. The reconstruction results are shown in **Figure 8**, and the quantitative indexes are shown in **Table 6**.

**Table 6:** Quantitative evaluation results of the CLT pseudotumor *in vivo* experiments.

Method	Reconstructed center/mm	*E_L_*/mm	*Dice*	*R_V_*
Tikhonov	15.67, 15.65, 19.83	4.91	0	1.33
P-EDDRS+Tikhonov	18.97, 18.51, 19.51	0.78	0.51	1.69
DSVD	18.63, 14.81, 18.55	3.80	0	1.11
P-EDDRS+DSVD	19.28, 18.16, 19.95	0.89	0.50	1.09

CLT, Cerenkov luminescence tomography; P-EDDRS, probabilistic energy distribution density region scaling; DSVD, damped singular value decomposition.

In **Figure 8**, the radiation source is marked in red, and the reconstruction source distribution is shown in a purple grid in all 3D views. In all axial views, the actual location of the radiation source is marked by a red arrow. As shown in **Figures 8C, D**, Tikhonov and DSVD algorithms in one-step reconstruction cannot obtain the correct radiation source depth in the *in vivo* CLT experiment. In addition, it can be seen from **Figures 8C, D** that Tikhonov and DSVD algorithms identify multiple radiation source distribution. However, in **Figures 8E, F**, the P-EDDRS framework can ensure that the distribution of the reconstructed radioactive sources tends to be a whole, and the center position of the reconstructed radioactive sources with the P-EDDRS framework was relatively close to the actual pseudotumor model. According to the quantitative results in **Table 6**, the *E_L_* of all results is between 0.78 and 0.89, and the Dice coefficient directly rises from 0 in the initial result to more than 50%.

#### 3.2.2 The Experiment of Subcutaneous Breast Cancer

**Figure 9:**
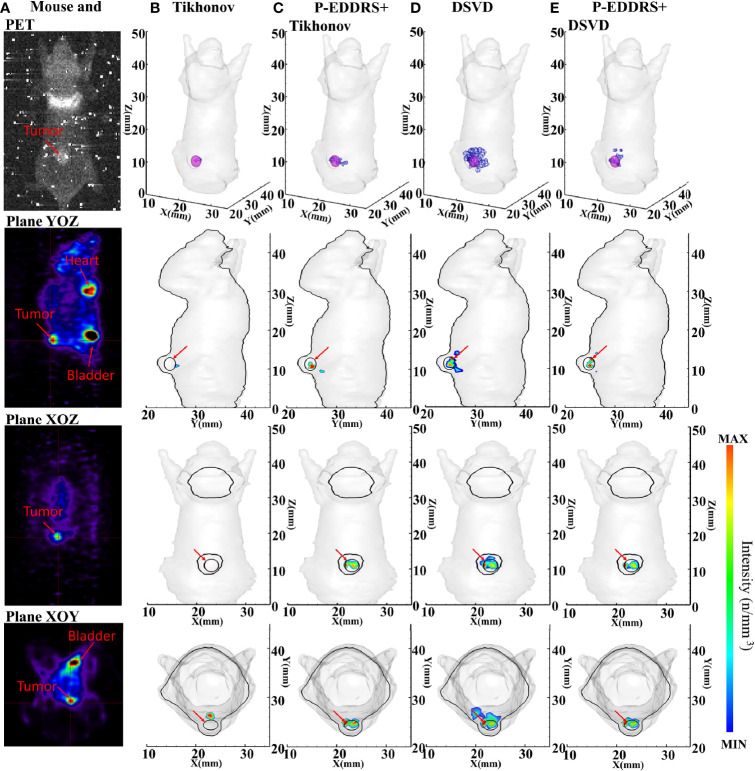
Results of the CLT subcutaneous breast experiments. All results include 3D views and corresponding cross-section views at the plane of X = 22.7, Y = 24.4 mm, and Z = 11 mm. **(A)** The image collected by the CCD camera; and PET results in YOZ, XOZ, and XOY planes. **(B, D)** The initial reconstruction results of Tikhonov and DSVD algorithms, respectively. **(C, E)** The results obtained by the corresponding algorithms of panels **(B, D)** after using our framework. CLT, Cerenkov luminescence tomography; DSVD, damped singular value decomposition.

In this experiment, a mouse implanted with 4T1 breast cancer cells was used as the target animal. The optical parameters, equipment, and experiment setting are the same as in *Section 3.2.1*. Special thanks to the Institute of Automation, Chinese Academy of Sciences for the data provided. The mouse was dispersed into a grid of 14,289 nodes and 71,838 tetrahedrons using FEM. The difference is that this experiment used the subcutaneous tumor and is not influenced by other tissues and organs. Therefore, to reduce the computational complexity, the tissues and organs inside the mouse were removed, and the mouse was regarded as a homogeneous structure composed of muscle tissues; 1 × 10^6^ 4T1 cells were subcutaneously injected into the back of the mouse. After 6 days of culture, about 800 ± 50 μCi of ^18^F-FDG was injected through the tail vein as a radioactive source. After 40 min, a CCD camera was used, the exposure time was 5 min, and the image in **Figure 9A** was collected. The Tikhonov and DSVD algorithms mentioned above were used to reverse reconstruct it in combination with the framework. In this experiment, the approximate central coordinates of the tumor are (22.7, 24.4, 11.0) mm. Breast cancer tumor was used as the focus in this experiment, and PET is currently the gold standard for imaging tumors, so PET data were introduced as a reference in **Figure 9A**. The figure of collected data, approximate tumor location obtained by PET, and reconstruction results are shown in **Figure 9**, and quantitative indicators are shown in **Table 7**.

**Table 7:** Quantitative evaluation results of the CLT subcutaneous breast experiments.

Method.	Reconstructed center/mm.	*E_L_*/mm.	*Dice.*	*R_V_*
Tikhonov	22.54, 26.36, 10.75	1.98	0.06	10.07
P-EDDRS+Tikhonov	23.53, 24.95, 10.96	0.99	0.46	1.84
DSVD	23.61, 25.35, 12.45	1.83	0.21	0.43
P-EDDRS+DSVD	23.57, 24.79, 10.94	0.69	0.43	1.57

CLT, Cerenkov luminescence tomography; P-EDDRS, probabilistic energy distribution density region scaling; DSVD, damped singular value decomposition.

In **Figure 9**, the radiation source is marked in red, and the reconstruction source distribution is shown in a purple grid in all 3D views. In all axial views, the actual location of the radiation source is marked by a red arrow. PET scan results were compared in this experiment to better compare with the actual situation, as shown in **Figure 9A**. It can be seen from **Figure 9A** that the noise signal collected in CLT contains many high-energy particles and thermal noise caused by prolonged exposure. This makes noise reduction of CLT particularly important and necessary. Therefore, this paper introduces the FLICMCDD denoising algorithm to preprocess the collected signals (34).

It can be seen from **Figure 9B** that the radiation source distribution obtained by Tikhonov algorithm in a single calculation is too sparse, with only a few nodes. The same conclusion can be reached in the volume ratio of quantitative results. Compared with Tikhonov algorithm, the radiation source distribution obtained by DSVD algorithm is larger than the actual tumor, because the noise obtained by this group of data is too large, resulting in severe artifacts. The comparison of **Figures 9B–E** shows better results. According to the quantitative results, Tikhonov and DSVD algorithms have improved radiation source positioning accuracy, size, and shape using this paper’s framework in CLT. From all the results, the *E_L_* of all the results stays below 1. However, artifacts appeared in the results of different methods. This is because the grid of our group is too simplified and ignores the influence of other tissues of mouse on CR, which ultimately leads to a more significant distortion of the collected CR signal. This phenomenon also leads to a low Dice coefficient compared with numerical simulation experiments. However, it can be seen from the comparison of **Figures 9D, E** that the framework has a specific elimination effect on artifacts. This phenomenon happens because the reconstruction results with artifacts have a sizeable error rate, which leads to a significant decrease in their weight. In the final result, the intensity will be weakened accordingly, reducing the impact on the overall result, which will be shown as weak artifacts from the cross-section diagram. On the whole, the reconstructed radioactive source distribution in **Figures 9C, E** can be consistent with PET.

## 4 Discussion and Conclusions

This paper proposed a multilevel ROI-scaled CLT reconstruction framework based on probabilistic energy density. In this framework, all nodes with energy are regarded as random variables of 3D spatial distribution, and the cuboid ROI region is used to ensure spatial continuity between nodes. Optimize the defect of neglecting the spatial distribution of nodes in the iterative reconstruction method only based on energy intensity. In order to ensure a stable ROI regional change rate, a shrinking formula based on energy intensity is introduced to ensure that energy information and spatial information of nodes are considered simultaneously. By the idea of iterative reconstruction, the corresponding probability value is assigned to the result of each reconstruction and dynamically refreshed in each iteration to avoid the contamination of the global result by some iteration errors such as radioactive source is too sparse and scattered or the artifact is too prominent in CLT.

To verify and evaluate the feasibility and performance of the proposed framework in CLT, numerical simulation and *in vivo* experiments are introduced. Four reconstruction algorithms, Tikhonov, DSVD, LassoLSQR, and OMP, were used for qualitative and quantitative analyses and comparison. The following conclusions can be obtained from the experimental results: first, the feasibility experiment shows that our framework can improve the radioactive source positioning accuracy of different algorithms in CLT; all *E_L_* values stay below 1; second, our frame has advantages in positioning accuracy and radioactive source shape recovery ability for CLT compared with other one-step reconstruction or iterative reconstruction method, the Dice coefficient of all numerical simulation experiments is above 0.65 and above 0.43 *in vivo* experiments; third, the experimental results of different sizes and shapes of radioactive sources show that our frame is robust and can better obtain the size and shape of radioactive sources for CLT; finally, *in vivo* experiments for CLT verified the feasibility of this framework in the detection of the radioactive source in live animals. It is worth noting that the reconstruction result of *in vivo* experiment is slightly worse than that of the numerical simulation experiment. This is because in *in vivo* experiments, the CCD camera collected a relatively weak CR signal and a large number of high-energy rays produced by radionuclides and thermal noise of the CCD camera itself. Furthermore, the deviation will inevitably occur in the process of energy mapping to the grid.

There are still some deficiencies in the framework. First of all, this framework is based on the idea of iterative multiple times. Compared with a one-step reconstruction, the calculation cost is high. Second, this framework is based on the finite element mesh method, resulting in inconsistent reconstruction performance for different shapes and sizes of radioactive sources. In addition, the cube ROI does not fit the irregular radioactive source well, leading to the low Dice coefficient in some cases. Subsequent work will attempt to reconstruct using other structures such as voxels or point clouds and try ROI of other shapes to overcome current deficiencies.

On the whole, the P-EDDRS frame treats the nodes resulting from each reconstruction as 3D random variables. According to the distribution characteristics of random variables, cuboid ROI was used to delimit molecular regions to ensure the spatial continuity of reconstruction results. Shrinking formula based on energy intensity is introduced to ensure that energy node information and spatial information jointly control regional ROI changes. Dynamic probabilistic results can guarantee the correctness of reconstruction results. The reconstruction results of numerical simulations and *in vivo* experiments demonstrate that our framework can improve the ability of multiple reconstruction algorithms to locate the radioactive source and recover the shape of the radioactive source.

## Data Availability Statement

The raw data supporting the conclusions of this article will be made available by the authors, without undue reservation.

## Ethics Statement

The animal study was reviewed and approved by Animal Ethics Committee of the Northwest University of China (No. NWU-AWC-20210901M).

## Author Contributions

XW: conceptualization, methodology, software, programing, validation, visualization, data curation, writing—original draft, and writing—review and editing. HG: conceptualization, writing—review and editing, funding acquisition, and data curation. JY: writing—review and editing, and funding acquisition. XLH: conceptualization and writing—review and editing. HY: funding acquisition. YH: project administration and funding acquisition. XWH: writing—review and editing, supervision, project administration, and funding acquisition. All authors contributed to the article and approved the submitted version.

## Funding

This study was funded by the National Natural Science Foundation of China under Grants 61971350, 61901374, 61906154, and 11871321; Natural Science Foundation of Shaanxi under Grant 2019JQ-724; Postdoctoral Innovative Talents Support Program under Grant BX20180254; Scientific and Technological projects of Xi’an under Grant 201805060ZD11CG44; Key Research and Development Program of Shaanxi 2020SF-036; Xi’an Science and Technology Project 2019218214GXRC018CG019-GXYD18.3; and Education Department Served Local Special Projects under Grant 16JF026.

## Conflict of Interest

The authors declare that the research was conducted in the absence of any commercial or financial relationships that could be construed as a potential conflict of interest.

## Publisher’s Note

All claims expressed in this article are solely those of the authors and do not necessarily represent those of their affiliated organizations, or those of the publisher, the editors and the reviewers. Any product that may be evaluated in this article, or claim that may be made by its manufacturer, is not guaranteed or endorsed by the publisher.

## References

[1] Weissleder R Pittet MJ. Imaging in the Era of Molecular Oncology. Nature (2008) 452(7187):580–9. doi: 10.1038/nature06917 PMC2708079 18385732

[2] Robertson R Germanos MS Li C Mitchell GS Cherry SR Silva MD. Optical Imaging of Cerenkov Light Generation From Positron-Emitting Radiotracers. Phys Med Biol (2009) 54(16):N355–65. doi: 10.1088/0031-9155/54/16/n01 PMC2765256 19636082

[3] Cao X Wei X Yan F Wang L Su L Hou Y. A Novel Stacked Denoising Autoencoder-Based Reconstruction Framework for Cerenkov Luminescence Tomography. IEEE Access (2019) 7:85178–89. doi: 10.1109/access.2019.2924042

[4] Klein JS Mitchell GS Stephens DN Cherry SR. Theoretical Investigation of Ultrasound-Modulated Cerenkov Luminescence Imaging for Higher-Resolution Imaging in Turbid Media. Optics Lett (2018) 43(15):3509–12. doi: 10.1364/ol.43.003509 PMC6192031 30067696

[5] Wei X Lu D Cao X Su L Wang L Guo H. A Fuzzy Artificial Neural Network-Based Method for Cerenkov Luminescence Tomography. Aip Adv (2019) 9(6):065105. doi: 10.1063/1.5088234

[6] Darr C Krafft U Fendler WP Costa PF Barbato F Praus C. First-In-Man Intraoperative Cerenkov Luminescence Imaging for Oligometastatic Prostate Cancer Using Ga-68-PSMA-11. Eur J Nucl Med Mol Imaging (2020) 47(13):3194–5. doi: 10.1007/s00259-020-04778-y 32356006

[7] Geng C Ai Y Tang X Shu D Gong C Guan F. A Monte Carlo Study of Pinhole Collimated Cerenkov Luminescence Imaging Integrated With Radionuclide Treatment. Australas Phys Eng Sci Med (2019) 42(2):481–7. doi: 10.1007/s13246-019-00744-7 30830649

[8] Park JC An GI Park S-I Oh J Kim HJ Ha YS. Luminescence Imaging Using Radionuclides: A Potential Application in Molecular Imaging. Nucl Med Biol (2011) 38(3):321–9. doi: 10.1016/j.nucmedbio.2010.09.003 21492780

[9] Kothapalli S-R Liu H Liao JC Cheng Z Gambhir SS. Endoscopic Imaging of Cerenkov Luminescence. Biomed Optics Express (2012) 3(6):1215–25. doi: 10.1364/boe.3.001215 PMC3370963 22741069

[10] Thorek DLJ Ogirala A Beattie BJ Grimm J. Quantitative Imaging of Disease Signatures Through Radioactive Decay Signal Conversion. Nat Med (2013) 19(10):1345. doi: 10.1038/nm.3323 24013701 PMC3795968

[11] Li C Mitchell GS Cherry SR. Cerenkov Luminescence Tomography for Small-Animal Imaging. Optics Lett (2010) 35(7):1109–11. doi: 10.1364/ol.35.001109 PMC2852688 20364233

[12] Guo H Hu Z He X Zhang X Liu M Zhang Z. Non-Convex Sparse Regularization Approach Framework for High Multiple-Source Resolution in Cerenkov Luminescence Tomography. Optics Express (2017) 25(23):28068–85. doi: 10.1364/oe.25.028068

[13] Ding X Wang K Jie B Luo Y Hu Z Tian J. Probability Method for Cerenkov Luminescence Tomography Based on Conformance Error Minimization. Biomed Optics Express (2014) 5(7):2091–112. doi: 10.1364/boe.5.002091 PMC4102351 25071951

[14] Cai M Zhang Z Shi X Yang J Hu Z Tian J. Non-Negative Iterative Convex Refinement Approach for Accurate and Robust Reconstruction in Cerenkov Luminescence Tomography. IEEE Trans Med Imaging (2020) 39(10):3207–17. doi: 10.1109/tmi.2020.2987640 32324543

[15] Hu Z Liu M Zhang Z Guo H Tian J. A Novel Radiopharmaceutical-Excited Fluorescence Tomography of the Mice Bearing Hepatocellular Carcinoma. J Nucl Med (2016) 57(supplement 2):1421.

[16] Ciarrocchi E Belcari N. Cerenkov Luminescence Imaging: Physics Principles and Potential Applications in Biomedical Sciences. Ejnmmi Phys (2017) 4(1):14. doi: 10.1186/s40658-017-0181-8 28283990 PMC5346099

[17] Spinelli AE Kuo C Rice BW Calandrino R Marzola P Sbarbati A. Multispectral Cerenkov Luminescence Tomography for Small Animal Optical Imaging. Optics Express (2011) 19(13):12605–18. doi: 10.1364/oe.19.012605 21716501

[18] Zhong J Qin C Yang X Zhu S Zhang X Tian J. Cerenkov Luminescence Tomography for *In Vivo* Radiopharmaceutical Imaging. Int J Biomed Imaging (2011) 2011(7):641618–8. doi: 10.1155/2011/641618 PMC3124671 21747821

[19] Guo H He X Liu M Zhang Z Hu Z Tian J. Weight Multispectral Reconstruction Strategy for Enhanced Reconstruction Accuracy and Stability With Cerenkov Luminescence Tomography. IEEE Trans Med Imaging (2017) 36(6):1337–46. doi: 10.1109/tmi.2017.2658661 28182554

[20] Cai TT Wang L. Orthogonal Matching Pursuit for Sparse Signal Recovery With Noise. IEEE Trans Inf Theory (2011) 57(7):4680–8. doi: 10.1109/tit.2011.2146090

[21] Yu J Zhang B Iordachita II Reyes J Lu Z Brock MV. Systematic Study of Target Localization for Bioluminescence Tomography Guided Radiation Therapy. Med Phys (2016) 43(5):2619–29. doi: 10.1118/1.4947481 PMC4859833 27147371

[22] Yi H Jiao P Li X Peng J He X. Three-Way Decision Based Reconstruction Frame for Fluorescence Molecular Tomography. J Optical Soc America a-Optics Image Sci Vision (2018) 35(11):1814–22. doi: 10.1364/josaa.35.001814 30461839

[23] Naser MA Patterson MS. Improved Bioluminescence and Fluorescence Reconstruction Algorithms Using Diffuse Optical Tomography, Normalized Data, and Optimized Selection of the Permissible Source Region. Biomed Optics Express (2011) 2(1):169–84. doi: 10.1364/boe.2.000169 PMC3028492 21326647

[24] Guo H Zhao H Yu J He X He X Song X. X-Ray Luminescence Computed Tomography Using a Hybrid Proton Propagation Model and Lasso-LSQR Algorithm. J Biophotonics (2021) 15:e202100089. doi: 10.1002/jbio.202100089 34176239

[25] Tikhonov AN Goncharsky AV Stepanov VV Yagola AG. Numerical Methods for the Solution of Ill-Posed Problems. Amsterdam: Springer Netherlands (1995). doi: 10.1007/978-94-015-8480-7

[26] Ekstrom MP Rhoads RL. On the Application of Eigenvector Expansions to Numerical Deconvolution. J Comput Phys (1974) 14(4):319–40. doi: 10.1016/0021-9991(74)90016-3

[27] Lv Y Tian J Cong W Wang G Luo J Yang W. A Multilevel Adaptive Finite Element Algorithm for Bioluminescence Tomography. Optics Express (2006) 14(18):8211–23. doi: 10.1364/oe.14.008211 19529195

[28] Dogdas B Stout D Chatziioannou AF Leahy RM. Digimouse: A 3D Whole Body Mouse Atlas From CT and Cryosection Data. Phys Med Biol (2007) 52(3):577–87. doi: 10.1088/0031-9155/52/3/003 PMC3006167 17228106

[29] Ren S Chen X Wang H Qu X Wang G Liang J. Molecular Optical Simulation Environment (MOSE): A Platform for the Simulation of Light Propagation in Turbid Media. PloS One (2013) 8(4):e61304. doi: 10.1371/journal.pone.0061304 23577215 PMC3620115

[30] Agostinelli S Allison J Amako K Apostolakis J Araujo H Arce P. GEANT4-A Simulation Toolkit. Nucl Instruments Methods Phys Res Section a-Accelerators Spectrometers Detectors Associated Equip (2003) 506(3):250–303. doi: 10.1016/s0168-9002(03)01368-8

[31] Pagliazzi M Ciarrocchi E Del Guerra A Belcari N Boschi F Spinelli AE. Development of a Simulation Environment for Cerenkov Luminescence Imaging. In: 60th IEEE Nuclear Science Symposium (NSS) / Medical Imaging Conference (MIC) / 20th International Workshop on Room-Temperature Semiconductor X-Ray and Gamma-Ray Detectors. Seoul: IEEE (2013). p. 1–6. doi: 10.1109/nssmic.2013.6829367

[32] Ackerman NL Boschi F Spinelli AE. Monte Carlo Simulations Support non-Cerenkov Radioluminescence Production in Tissue. J Biomed Optics (2017) 22(08):1. doi: 10.1117/1.jbo.22.8.086002 28819962

[33] Naser MA Patterson MS. Bioluminescence Tomography Using Eigenvectors Expansion and Iterative Solution for the Optimized Permissible Source Region. Biomed Optics Express (2011) 2(11):3179–92. doi: 10.1364/boe.2.003179 PMC3207385 22076277

[34] Cao X Sun Y Kang F Wang L Yi HJ Zhao FJ. A Novel Denoising Framework for Cerenkov Luminescence Imaging Based on Spatial Information Improved Clustering and Curvature-Driven Diffusion. J Innovative Optical Health Sci (2018) 11(4):8. doi: 10.1142/s1793545818500177

